# Molecular characterization of LMW-GS genes in *Brachypodium distachyon* L. reveals highly conserved *Glu-3* loci in *Triticum* and *related* species

**DOI:** 10.1186/1471-2229-12-221

**Published:** 2012-11-21

**Authors:** Shunli Wang, Ke Wang, Guanxing Chen, Dongwen Lv, Xiaofeng Han, Zitong Yu, Xiaohui Li, Xingguo Ye, SLK Hsam, Wujun Ma, Rudi Appels, Yueming Yan

**Affiliations:** 1 Key Laboratory of Genetics and Biotechnology, College of Life Science, Capital Normal University, 100048, Beijing, China; 2 Institute of Crop Sciences, Chinese Academy of Agricultural Sciences/National Key Facility for Crop Gene Resources and Genetic Improvement, 100081, Beijing, China; 3 Division of Plant Breeding and Applied Genetics, Technical University of Munich, D-85350, Freising-Weihenstephan, Germany; 4 State Agriculture Biotechnology Centre, Murdoch University; Western Australian Department of Agriculture and Food, Perth, WA, 6150, Australia

## Abstract

**Background:**

*Brachypodium distachyon* L. is a newly emerging model plant system for temperate cereal crop species. However, its grain protein compositions are still not clear. In the current study, we carried out a detailed proteomics and molecular genetics study on grain glutenin proteins in *B. distachyon*.

**Results:**

SDS-PAGE and RP-HPLC analysis of grain proteins showed that *Brachypodium* has few gliadins and high molecular weight glutenin subunits. In contrast the electrophoretic patterns for the albumin, globulin and low molecular weight glutenin subunit (LMW-GS) fractions of the grain protein were similar to those in wheat. In particular, the LMW-C type subunits in *Brachypodium* were more abundant than the equivalent proteins in common wheat. Southern blotting analysis confirmed that *Brachypodium* has 4–5 copies of LMW-GS genes. A total of 18 LMW-GS genes were cloned from *Brachypodium* by allele specific PCR. LMW-GS and 4 deduced amino acid sequences were further confirmed by using Western-blotting and MALDI-TOF-MS. Phylogenetic analysis indicated that *Brachypodium* was closer to *Ae. markgrafii* and *Ae. umbellulata* than to *T. aestivum*.

**Conclusions:**

*Brachypodium* possessed a highly conserved *Glu-3* locus that is closely related to *Triticum* and related species. The presence of LMW-GS in *B. distachyon* grains indicates that *B. distachyon* may be used as a model system for studying wheat quality attributes.

## Background

Cereals are the main cultivated crops in agriculture, including rice (*Oryza sativa* L.), wheat (*Triticum aestivum* L.), barley (*Hordeum vulgare* L.), rye (*Secale cereale* L.), oats (*Avena sativa* L.), maize (*Zea ma*ys L.) and sorghum (*Sorghum bicolor* L.). They belong to the family of the Poaceae. The major grain proteins in cereal crops are storage proteins, accounting for about 60-80% of total proteins depending on species and varieties. Prolamins are major seed storage proteins in wheat, barley, rye and maize, and are important nutrition and food sources for both humans and animals. Globulins are the predominant storage proteins in oat and rice, accounting for about 70-80% of the total seed proteins [1]. Previous studies have been largely focused on wheat, rice, maize and sorghum due to their importance as food crops in the world. In wheat the major seed storage proteins include glutenins and gliadins that are the primary determinations of dough elasticity and extensibility, respectively [2], and both protein groups play a key role in the processing of wheat flour into different baked products.

So far, many prolamin genes have been cloned, not only in common wheat but also in its related species such as *Aegilops* L., *Agropyron cristatum*, *Thinopyrum intermedium*, *Lophopyrum elongatum*, *Dasypyrum villosum*, *Crithopsis delileana*, *Eremopyrum distans,* and *Taeniatherum caput-medusae*[3-10]. The major protein components in milled rice or rice endosperm were glutenins, which are different from the prolamins in other cereals [11]. Rice (*Oryza sativa* L.) as a major crop with a relatively small genome (about 451 Mbp) has been promoted as a model system of monocots [12]. However, its relatively longer life cycle, large physical stature and special semiaquatic growth requirement limited its wide usage as a model system. *Brachypodium distachyon* L. (*B. distachyon*), a member of the Pooideae subfamily and a temperate wild annual grass endemic to the Mediterranean and Middle East [13], has been rapidly established as a model plant system especially as an experimental model organism for grasses and cereals. It possesses many attractive attributes such as small genome (diploid with about 355 Mbp), short growth cycle, self-fertility and simple growth requirements [13,14], as well as competence to be efficiently transformed [15-17]. Furthermore the *B. distachyon* genome exhibits a high level of colinearity and synteny to the genomes of temperate cereal crops [18]. Thus, it has facilitated a range of studies in comparative genomics of cereals, including wheat, rice, and even *Aegliops* species.

Phylogenic analysis has classified *B. distachyon* to be closer to wheat and barley than to rice, corn or sorghum [19,20]. An efficient transformation procedure and an optimized plant regeneration protocol have been developed for *B. distachyon*, including conditions for inducing embryogenic callus [21] and biolistic transformation [15,16]. Vogel and Hill [22] identified an inbred diploid line, Bd21-3, with high transformation efficiencies. Despite these substantial studies and accumulated knowledge, its grain proteins and compositions are still not clear. Laudencia-Chingcuanco and Vensel [23] demonstrated that globulins are the main seed storage proteins in *B. distachyon* based on sodium dodecyl sulphate polyacrylamide gel electrophoresis (SDS-PAGE) and mass spectrometry (MS) analyses in a diploid accession Bd21. Larré *et al*. [24] found that globulins and a few prolamins in Bd21-3, a diploid inbred line originated from Bd21. Wang *et al*. [25] identified 18 storage proteins and 15 albumin proteins in Bd21, including a high molecular weight glutenin subunit (HMW-GS). However, so far low molecular weight glutenin subunits (LMW-GS) and their gene organizations in *B. distachyon* remain unknown although the genome sequencing of *B. distachyon* is completed.

In this work, LMW-GS in *B. distachyon* were separated and characterized by a proteomic approach and their encoding genes were isolated by allele specific PCR (AS-PCR). A phylogenetic analysis among cereal crops was also carried out. Our results revealed that a highly conserved *Glu-3* locus is present in *B. distachyon* being similar to that in *Triticum* and *Aegilops* species.

## Results

### Separation and characterization of *B. distachyon* grain proteins

**Figure 1:**
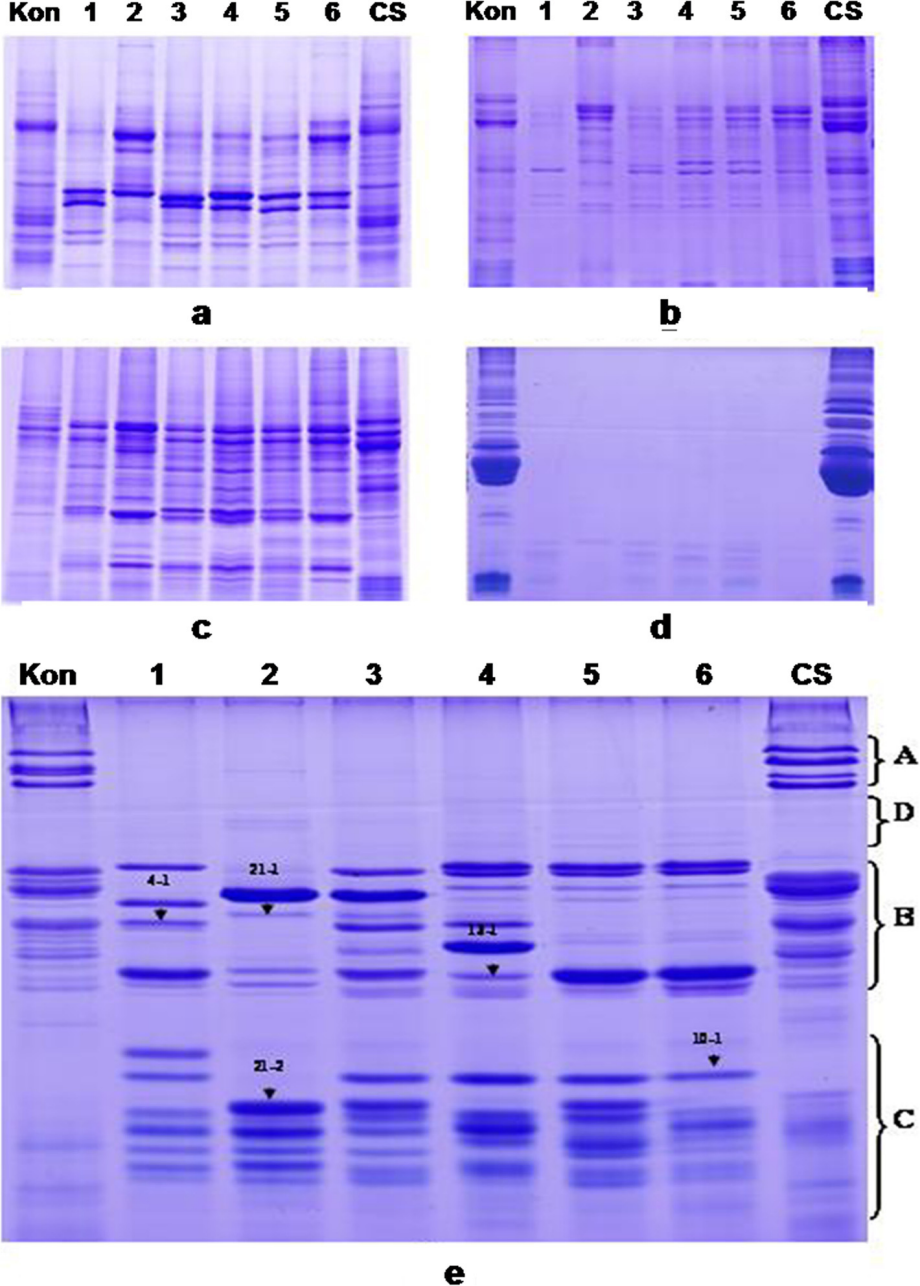
**Separation and characterization of grain proteins from 6 *Brachypodium *accessions and common wheat varieties Chinese Spring (CS) and Kontrast (Kon) by SDS-PAGE.** The sample number of lane 1–6 from left to right was *Brachypodium* accession Bd4 (PI208216), Bd21, Bd16 (PI239715), Bd13 (PI233228), Bd11 (PI226629) and Bd10 (PI226452), respectively. **a**. Albumins; **b**. Globulins; **c**. Albumins and globulins; **d**. Gliadins; **e**. LMW glutenin subunits. Different glutenin groups were indicated by A (HMW-GS), B (B-type LMW-GS), C (C-type LMW-GS) and D (D-type LMW-GS), respectively. Arrows indicate 5 representative LMW-GS bands (4–1, 21–1, 21–2, 13-1and 16–1) selected for further identification by MALDI-TOF-MS.

The grain protein compositions of 6 accessions of *B. distachyon* (Bd4, Bd10, Bd11, Bd13, Bd16 and Bd21) and 2 common wheat cultivars (Chinese Spring and Kontrast) were analysed. The protein fractions analyzed included albumins, globulins, gliadins and glutenins. The results showed that the SDS electrophoresis patterns of albumins and globulins in *B. distachyon* accessions as well as their overall amount were generally similar to those of common wheat although there were differences in certain protein subunits and expression levels (Figure 1a-c). In contrast, the gliadins, HMW-GS and LMW-GS compositions displayed clear differences between *B. distachyon* and wheat. Only a few gliadin bands in *B. distachyon* were present in the lower molecular weight area on SDS-PAGE gels as shown in Figure 1d. Based on Figure 1e, the *B. distachyon* accessions had B and C but very few A and D type subunits, suggesting that *B. distachyon* accessions possess few HMW glutenin subunits. This result was similar to the previous report [25]. Meanwhile, *B. distachyon* accessions had similar LMW glutenin compositions to these of common wheat. The LMW C-type subunits were much more prominent than those of common wheat in both subunit components and expression levels. At least 9 distinct bands of each accession were visualized after staining on the SDS-PAGE gel.

**Figure 2:**
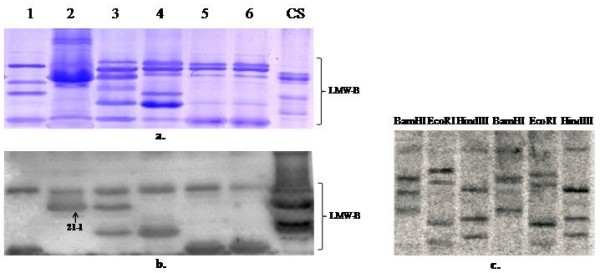
**Identification of LMW-GS of *B. distachyon *by Western blotting and Southern blotting.a**. The gel stained by Coomassie brilliant blue after Western blotting. **b**. Western blotting analysis of *B. distachyon*. The sample number of lane 1–6 from left to right was *Brachypodium accession* Bd4 (PI208216), Bd21, Bd16 (PI239715), Bd13 (PI233228), Bd11 (PI226629) and Bd10 (PI226452), respectively. **c**. Southern blotting analysis of Bd21. Genomic DNA from Bd21 was digested with BamHI, EcoRI and HindIII restriction enzymes.

In order to confirm our results, LMW-GS in *B. distachyon* were extracted with a wheat glutenin extraction method and separated by SDS-PAGE, and then transferred to PVDF membrane for Western-blotting and antibody-based identification. As shown in Figure 2, some positive signals could be observed for the LMW-B bands of both CS and *B. distachyon*, indicating the presence of LMW-GS in *B. distachyon*. Further Southern blotting analysis, using wheat LMW-GS gene as probe, demonstrated that the copies of LMW-GS genes in diploid *Brachypodium distachyon* 21 were 4–5 (Figure 2c).

**Figure 3:**
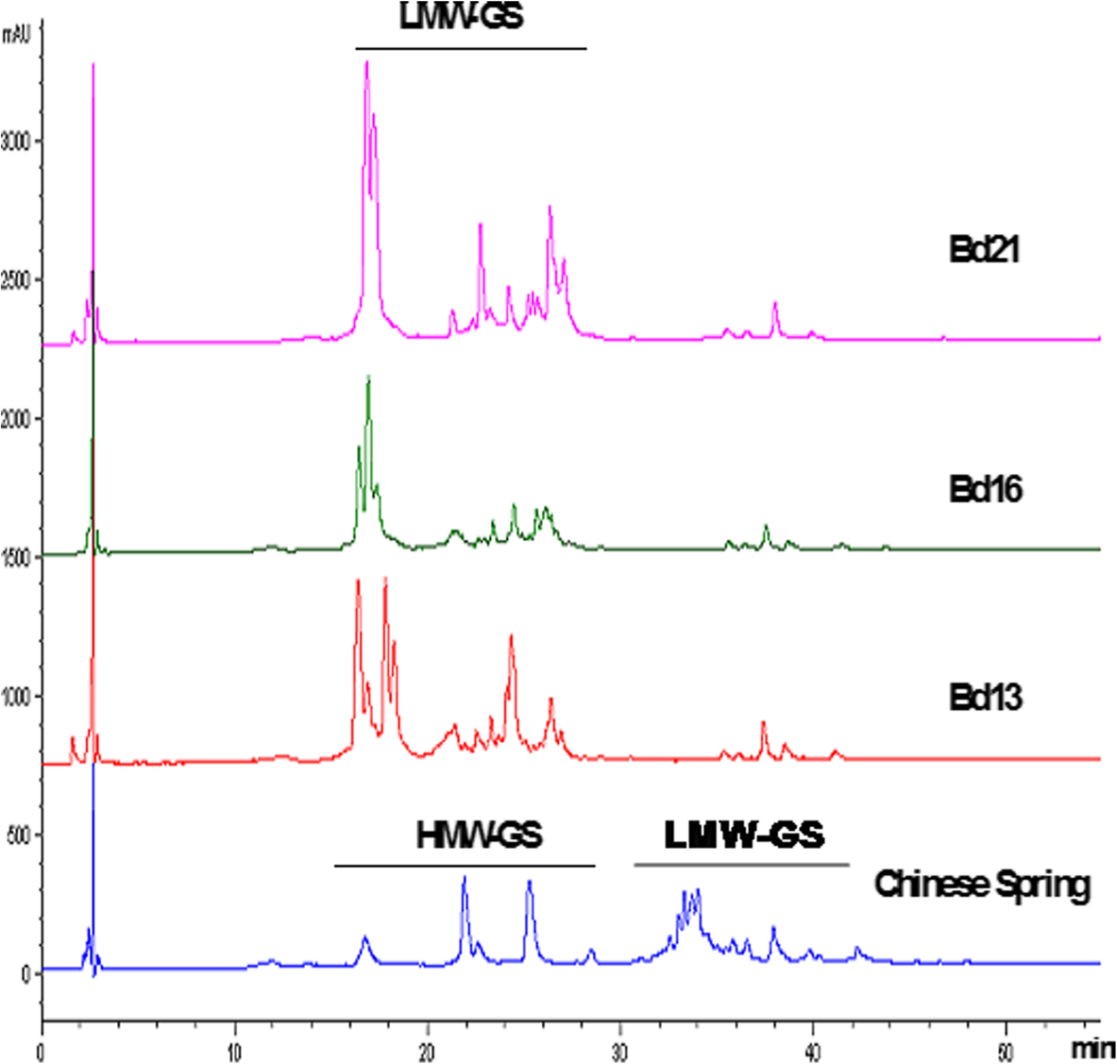
**Separation of glutenin subunits from Bd21, Bd16, Bd13 and Chinese Spring by RP-HPLC.** HMW-GS and LMW-GS were indicated.

The results of RP-HPLC analysis from Bd21, Bd13 and Bd16 as well as Chinese Spring are shown in Figure 3. It is known that proteins are separated by RP-HPLC according to their surface hydrophobicities and proteins with higher hydrophobicities elute faster than those with lower hydrophobicities. HMW-GS have been shown to have higher surface hydrophobicities and therefore elute earlier than LMW-GS [26]. As shown in Figure 3, HMW-GS and LMW-GS of Chinese Spring eluted at 15–30 min and 30–45 min, respectively. Interestingly, the LMW-GS from *B. distachyon* had similar elution time of HMW-GS from Chinese Spring, suggesting that they have similar hydrophobicities. Thus, although *Brachypodium* and common wheat had similar LMW-GS electrophoretic compositions, their protein structures and properties might be different.

### Molecular characterization of LMW-GS genes from *B. distachyon*

According to SDS-PAGE, Western blotting, Southern blotting and RP-HPLC analysis, *Brachypodium* has a homologous *Glu-3* locus that is similar to that in common wheat. Different AS-PCR primers for wheat LMW-GS genes were used to amplify the homologous genes from *B. distachyon*. In the current study, a total of 18 LMW-GS genes were amplified, cloned and sequenced from *B. distachyon*, including 12 LMW-m and 6 LMW-i type genes. The PCR amplification products on the agarose gel were showed in Additional file 1. All genes were deposited in GenBank (accession numbers in Table 1). Of the 18 genes cloned, 12 LMW-m type genes were from hexaploid *Brachypodium* while 6 LMW-i type genes were from diploid and hexaploid *Brachypodium* (HQ220189 and HQ220190 from Bd4, HQ220195 and HQ220197 from Bd10, HQ220191 and HQ220193 from Bd21)*.*

**Table 1:** **The cloned LMW-GS genes from *B. distachyon *L**

***B. distachyon *accessions**	**GenBank accession No.**	**Size (bp)**	**Deduced amino acid *M*r**	**Type**	**No. of Cysteine**
Bd4 (PI208216, 6n=30)	HQ220189	1170	42.678KD	LMW-i	8
	HQ220190	1095	39.839KD	LMW-i	8
Bd10 (PI226452, 6n=30)	HQ220192	897	31.748KD	LMW-m	8
	HQ220194	894	31.437KD	LMW-m	8
	HQ220196	885	31.223KD	LMW-m	8
	HQ220195	1038	37.723KD	LMW-i	8
	HQ220197	1035	37.568KD	LMW-i	8
Bd11(PI226629, 6n=30)	HQ220199	885	31.257KD	LMW-m	8
	HQ220201	885	31.279KD	LMW-m	8
	HQ220202	900	31.794KD	LMW-m	9
	HQ220203	885	31.333KD	LMW-m	8
	HQ220204	900	31.728KD	LMW-m	9
Bd13(PI 233228, 6n=30)	HQ220205	894	31.410KD	LMW-m	8
	HQ220206	885	31.229KD	LMW-m	7
Bd16(PI 239715, 6n=30)	HQ220198	894	31.377KD	LMW-m	8
	HQ220200	894	31.437KD	LMW-m	8
Bd21(PI 254867, 2n=10)	HQ220191	1164	42.551KD	LMW-i	8
	HQ220193	1134	41.360KD	LMW-i	8
*T. aestivum* cv. Chinese Spring	FJ615311	924	32.863KD	LMW-m	8
*T. aestivum* cv. Chinese Spring	AY453154	1131	41.266KD	LMW-i	8

**Figure 4:**
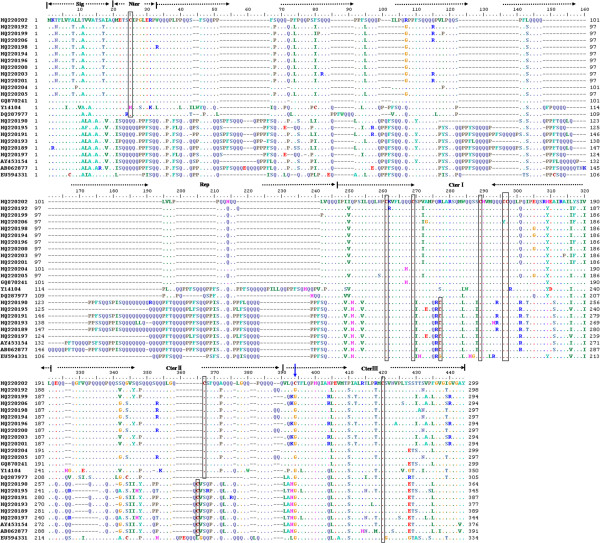
**Mutliple alignment of the deduced amino acid sequences of 24 LMW-GS genes, included 18 cloned from Brachypodium.** The other 6 genes were from wheat or *Aegliops* deposited in GenBank: Y14104, AY453154 and AB062877 from *T. turgidum subsp. durum*, *T. aestivum*, *T. aestivum*, and GQ870241, EU594331 and DQ287977 from *Ae. umbellulata*, *Ae. comosa*, *Ae. tauschii*, respectively. Black frames show the conserved positions of cysteine residues. Blue arrow showed the extra cysteine.

Comparative analysis of the nucleotide and the deduced protein sequences showed that all LMW-GS genes isolated from *B. distachyon* had the typical structural characteristics with those of common wheat and related species (Figure 4). The deduced protein sequences had a common structure model: a conserved 20 residues signal peptide, a 13 residues N-terminal domain (deletion in LMW-i type), a variable repetitive domain, and three subregions of the C-terminal domain. It is evident that *Brachypodium* had highly homologous *Glu-3* loci to those in *Triticum* and related species.

Sequence comparative analysis of 12 LMW-m and 6 LMW-i type genes showed a higher similarity. Thus, 5 typical LMW-m and LMW-i type genes were used to identify SNPs and InDels through comparison with 23 wheat LMW-GS genes from GenBank, and the results were showed in Table 2 and Table 3. For the 5 LMW-m type genes, a total of 34 SNPs were identified at different positions, and the numbers of SNPs in HQ220202, HQ220206, HQ220198, HQ220194, and HQ220200 were 4, 9, 8, 7 and 6, respectively. A total of 31 SNPs, accounting for 91.17%, were resulted from A→G or C→T transition, slightly higher than that of LMW-i type genes from *Triticum momoccocum*[27]. The remaining 3 SNPs (8.83%) were generated from T→A or C→A transition. Of the 34 SNPs detected, 22 variations belonged to nonsynonymous and 12 were synonymous mutations. No InDels were found among the 5 LMW-m type genes. For the 5 LMW-i type genes, only 9 SNPs were detected (Table 3), of which 7 were nonsynonymous substitutions. Both HQ220195 and HQ220191 had a synonymous substitution. In particular, HQ220190 had no SNPs, but contained 6 deletions at the position 346 (C), 349–350 (CA), 352–353 (CA), 355 (C), 391 (T), 397–404 (CAACAACA). All nucleotide acid deletions encoded a glutamine residue, except for the position 391 that encoded a serine.

**Table 2:** The positions of SNPs and InDels identified among 5 LMW-m type genes

LMW-GS	22	97	108	582	669	722	749	760	780	785	801	843	905	1033	1055	1058	1072	1078	1121	1135	1142
HQ220202	G	T	A	C	T	G	A	C	C	A	A	A	A	T	A	C	C	G	T	A	T
HQ220206	A	T	A	T	T	A	A	T	C	T	G	A	G	T	G	T	T	A	C	A	C
HQ220198	A	A	A	T	T	G	G	T	T	A	G	A	G	C	A	T	T	G	T	G	T
HQ220194	A	T	G	T	A	G	G	T	T	A	G	A	G	T	A	T	T	G	T	A	T
HQ220200	A	T	A	T	T	G	G	T	T	A	G	G	G	T	A	T	T	G	T	A	T
23 other LMW-m Genes*	G	T	A	T	T	G	A	T	C	A	A	A	A	T	A	T	T	G	T	A	T

*Other 23 LMW-m genes were from GenBank, including EU329425 (*Ae. markgrafii*), EU594338 (*Ae. comosa*), EU571721 and FJ824795 (*Ae. speltoides*), DQ287977 (*Ae. tauschii*), GQ870236 (*Ae. triuncialis*), FJ824801 (*Ae. bicornis*), EU571719 (*Ae. columnaris*), GQ389630 and GQ389629 (*Ae. cylindrica*), EF188286 (*Ae. geniculata*), GQ389632, EU305555 and EU189089 (*Ae. longissima*), GQ870241and EU571725 (*Ae. umbellulata*), FJ824799 (*Ae. searsii*), AB062852 (*T. aestivum*), Y14104 and EF188290 (*T. turgidum subsp.durum*), AY263369, AB062868 and FJ615311 (*T. aestivum*).

**Table 3:** The positions of SNPs and InDels identified among 5 LMW-i type genes*

LMW-GS	38	251	275	346	349-350	352-353	355	391	397-404	464	789	1116	1040	1117
HQ220190	C	A	A	-	--	--	-	-	--------	T	G	A	A	T
HQ220195	T	G	A	C	CA	CA	C	T	CAACAACA	T	G	G	A	C
HQ220191	C	A	G	C	CA	CA	C	T	CAACAACA	T	G	A	G	T
HQ220193	C	A	A	C	CA	CA	C	T	CAACAACA	C	C	A	A	T
HQ220197	C	A	A	C	CA	CA	C	T	CAACAACA	T	G	A	A	C
23 other														
LMW-m Genes	C	A	A	C	CA	CA	C	T	CAACAACA	T	G	A	A	T

*Horizontal dashes indicated the deletions of nucleotide. Other 23 LMW-i genes from GenBank included GQ870245 (*Ae. markgrafii*), GQ870242 (*Ae. umbellulata*), GQ870246 (*Ae. speltoides*), EU594331, EU594335 and EU594336 (*Ae. comosa*), EF536030 (*Ae. juvenalis*), EF536035 (*Ae. geniculata*), AY606257 and AY724436 (*L. elongatum*), GQ870247 (*T. urartu*), DQ217661 and DQ217663 (*T. turgidum subsp. dicoccoides*), DQ345449 (*T. monococcum* L.), AY453154, AY453155, AY453157, AY453160, EU189087, FJ549932, FJ755303, FJ755304 and AB062877 (*T. aestivum*).

Both HQ220202 and HQ220204 had an extra cysteine located at the 4^th^ residue in the C-terminal III domain, resulting from T→G dot mutation and leading to the cysteine generation from glycin. Additionally, HQ220206 only had 7 cysteine, and one cysteine residue was deleted in the C-terminal II domains, which was resulted from a G→A transition, and led to generate a tyrosine (TAT) from cysteine (TGT).

### LMW-GS determination by mass spectrometry

Mass spectrometry has shown to be effective in gaining structural information of glutenins and globulins directly isolated from seeds [23,28-30]. According to the deduced molecular masses (31-42kDa) of LMW-GS genes isolated from *Brachypodium* in this work, 5 representative protein bands on the SDS-PAGE (Figure 1e), corresponding to different molecular mass ranges, were chosen to further identify by MALDI-TOF-MS after trypsin digestion. The standard error (M+H)^+^ was based on Sun *et al*. [28] which was set to less than 3.212. The results of the mass spectrometric identifications were summarized in Table 4.

**Table 4:** **Protein identification from SDS-PAGE bands in *B. distachyon *by MALDI-TOF-MS**

**LMW-GS Genes**	**LMW-GS subunit**	**Measured mass (M+H)+**	**Calculated mass (M+H)+**	**Missed cleavage**	**Peptide sequences predicted by PeptideMass**	**Positions**
HQ220190	4-1	845.4112	842.4913	0	QTPEQSR	218-224
		1088.5128	1091.5411	0	QCCQQLR	211-217
		1704.7904	1707.8036	1	QCCQQLRQTPEQSR	211-224
		1765.9240	1765.7693	0	VFLQQQCIPVAMQR	179-192
		1793.8608	1794.8394	0	SQMLQQSICHVMQR	197-210
		2120.0925	2119.0759	1	VFLQQQCIPVAMQRCLAR	179-196
HQ220191	21-1	845.4112	842.5071	0	QTPEQSR	241-247
		878.3971	879.4393	0	QCCQQLR	234-240
		1704.7904	1707.8132	1	QCCQQLRQTPEQSR	234-247
		1765.9240	1765.7466	0	VFLQQQCIPVAMQR	202-215
		1793.8608	1791.7360	0	SQMLQQSICHVMQR	220-233
		2104.0976	2102.0032	1	VFLQQQCIPVAMQRCLAR	202-219
		2342.1501	2342.9780	1	CLARSQMLQQSICHVMQR	216-233
		2643.3044	2643.3130	0	MCSVNVPLYETTTSVPLGVG IGVGAY	342-367
HQ220195	13-1	1479.7662	1482.8090	1	QIPEQSRHESIR	202-213
		1687.9490	1687.8210	0	AIVYSIILQQQQQR	214-227
		1718.8716	1723.8390	0	VFLQQQCIPVEMQR	163-176
		1734.8665	1735.8500	0	VFLQQQCIPVEMQR	163-176
		1823.9295	1828.8950	0	VFLQQQCIPVEMQR	163-176
		2162.1031	2161.0060	1	VFLQQQCIPVEMQRCLAR	163-180
HQ220198	10-1	1593.7876	1595.7630	0	METSCIPGLERPR	1-13
		1850.9404	1851.8900	0	VFLQQQCSPIAMPQR	109-123
		2086.1048	2085.9630	1	VFLQQQCSPIAMPQRLAR	109-126
		2102.0997	2102.0290	1	VFLQQQCSPIAMPQRLAR	109-126
		3337.4388	3337.6920	0	SQMWQQSSCHVMQQQCCQQLQQIPGQSR	127-154
		3960.8448	3963.8230	1	LARSQMWQQSSCHVMQQQCCQQLQQIPGQSR	124-154

The calculated mass spectra of trypsin digested 18 deduced LMW-GS were compared with 5 MALDI-TOF measured peptide mass spectra, four LMW-GS HQ220190, HQ220191, HQ220195 and HQ220198 were well matched with subunits 4–1, 21–1, 13–1 and 10–1, respectively (Table 4, Additional file 2, Additional file 3, Additional file 4 and Additional file 5), indicating that they are true native subunits in *Brachypodium* grains. The subunit 21–1 has been detected by the Western blotting (Figure 2), and thus the mass spectra results further confirmed that this band was LMW-GS. The remaining Band 21–2 was identified as prolamine (Additional file 6 and Additional file 7), which is likely to be a C-type LMW-GS, corresponding to α/β- and γ-gliadins modified in the number of cysteine residues [31].

### Phylogenetic evolutionary analysis among *B. distachyon*, *Triticum* and *Aegliops species*

**Figure 5:**
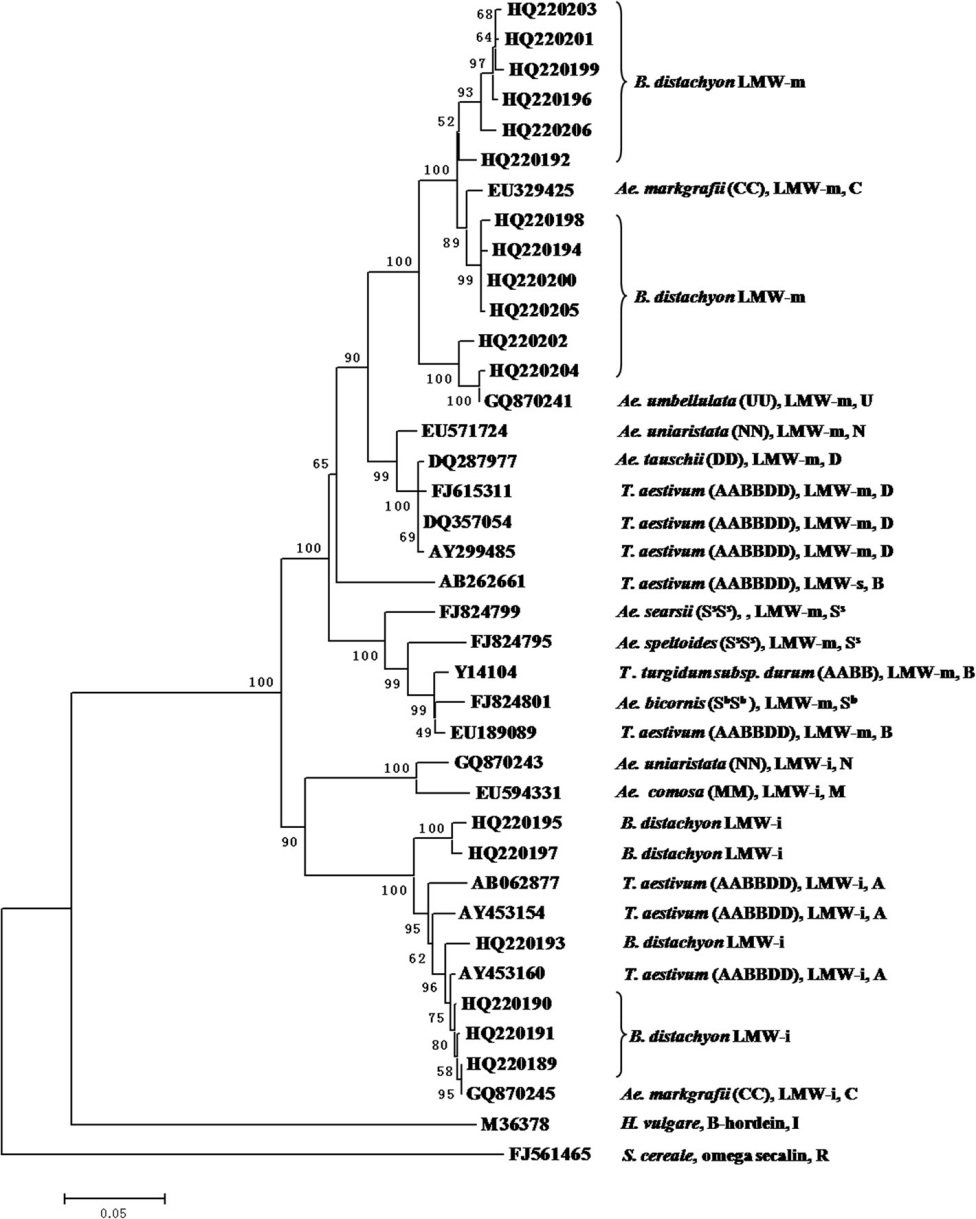
**the phylogenetic tree showed the relationships among *Brachypodium*, *Aegilops *and *Triticum *species based on the LMW-GS genes.** All LMW-GS genes cloned from *Brachypodium* were used to construct phylogenetic tree. Nucleotide sequences are specified by their accession number. A, B, or D at the end of the accession number indicates the chromosomal assignment of their sequences.

A total of 39 storage protein genes were used to construct phylogenetic trees and to investigate their evolutionary relationships among *Brachypodium*, *Aegliops*, and *Triticum* species as well as other cereal crops with complete nucleotide coding sequences. These storage protein genes included 18 LMW-GS genes from *B. distachyon* in this work, 8 and 11 LMW-GS genes from *Triticum* and related genomes (A, B, D, C, N, U, M, S^s^, S^b^) from the GenBank, respectively, and a B-hordein from barley and a secalin gene from rye (Figure 5). The results showed that 12 LMW-m and 6 LMW-i genes were tightly clustered into one branch. The omega-secalin gene from rye was obviously clustered into a separated group. The B-hordein gene from barley was clustered in a branch with the LMW-GS genes, but showing a significant difference in sequence homology. Among the LMW-GS gene subgroup, 2 clades corresponding to the LMW-m & s and LMW-i type genes were separated. The LMW-m genes from *B. distachyon* were clustered with *Ae. markgrafii* and *Ae. umbelluta* in the first clade while the LMW-i genes from *Brachypodium* were clustered with the A genome genes from *Triticum aestivum* and C genome of *Ae. markgrafii*. These results suggested that the LMW glutenin genes from *Brachypodium* were closer to *Aegilops* than to *Triticum* at the *Glu-3* loci.

## Discussion

### LMW-GS as important grain storage proteins in *B. distachyon*

Based on the solubility in a series of solvents, plant proteins are traditionally classified into water-soluble proteins (albumins), saline-soluble proteins (globulins) and alcohol-soluble proteins (prolamins) [1]. Different plant species generally have one predominant protein type in the grain endosperm. Laudencia-Chingcuanco and Vensel [23] reported that globulins were the main seed storage proteins in *B. distachyon*. But in their work, only 7 major protein bands were detected by SDS-PAGE and mass spectrometry, of which 6 were identified as globulins. Glutenins and gliadins were not extracted and analyzed. Recently, LMW-GS like proteins were identified by SDS-PAGE, and their masses were determined by MALDI-TOF in *B. distachyon*[25]. These suggest that *Brachypodium* grains may contain LMW-GS like proteins.

Our results demonstrated that *Brachypodium* grains had similar electrophoretic compositions with common wheat in LMW glutenin subunits, In particular, *B. distachyon* appeared to have more abundant LMW-C type subunits than wheat (Figure 1). A total of 18 LMW-GS genes isolated from *B. distachyon* displayed highly homologous with those from *Triticum* and *Aegilops* species. The presence of LMW-GS in *B. distachyon* was further confirmed by Western blotting, Southern blotting and MALDI-TOF-MS (Table 4, Figure 2 and Additional file 2, Additional file 3, Additional file 4, Additional file 5, Additional file 6 and Additional file 7). Glutenins play important roles in the plant life cycle [1]. Therefore, the primary role of LMW-GS in *B. distachyon* is probably involved in providing energy and nutrition for seed germination.

### Allelic variations and gene organization at *Glu-3* in *B. distachyon*

High homology of LMW-GS genes between *Brachypodium* and common wheat and related species was found in this work (Figure 4,5), including similar gene sizes and structural characteristics. This suggests that a highly conserved *Glu-3* locus is present in *B. distachyon.* Of the 18 LMW-GS genes isolated from *B. distachyon*, extensive allelic variations were detected, including 34 and 9 SNPs in 5 typical LMW-m and LMW-i type genes, respectively, and 6 deletions present in the LMW-i type gene HQ220190. Particularly, both HQ220202 and HQ220204 had an extra cysteine, locating at the same position as GQ870250 and GQ870241 from *Ae. markgrafii* and *Ae. umbellulata*, respectively [32] (Figure 4). Thus, they probably represent an ancient type among the LMW-GS gene family. On the other hand, as in previous reports [9], HQ220206 only had 7 cysteine residues and a cysteine in the C-terminal II domain was changed into tyrosine because of a G→A transition. Therefore, the abundant SNP and InDel variations present in the LMW-GS genes of *B. distachyon* could result in different biochemical properties of their deduced protein subunits such as higher surface hydrophobicity as revealed by RP-HPLC (Figure 3).

The LMW-m and LMW-i genes isolated from *B. distachyon* were used to blast at the *Brachypodium* genome project websites (http://www.phytozome.net and http://www.brachypodium.org/). The blast results only returned one locus, Bradi3g17070, which had some sequence similarity with the LMW-GS genes cloned in this work. However, Bradi3g17070 was more similar to gliadin or avenin-like seed proteins. This suggests that the data of the *Glu-3* locus encoding for LMW-GS in *B. distachyon* is not present in the genome sequence database.

So far, although considerable work was carried out, the precise gene organizations at the *Glu-3* loci in wheat and related species are still unclear. LMW-GS can be devided to LMW-s, LMW-m, and LMW-i types according to the first amino acid residue of the mature protein, serine, methionine, or isoleucine, respectively [33]. LMW-GS are encoded by multiple gene family at the *Glu-3* loci of A, B, D chromosomes of common wheat [33]. Some other related genomes, e.g. S, C, M, N and U also contain highly homologous *Glu-3* loci [8,9,32]. According to previous reports, the copy numbers of the LMW-GS genes in common wheat were estimated to be 10–15 [33,34] or 35–40 [35-37]. Wicker *et al*. [38] found that two LMW-i type genes from the A genome of *Triticum monococcum* that were located more than 150 kbp apart. This could facilitate the occurrence of illegitimate recombination events within the LMW-GS genes and result in novel allelic variations, such as chimeric genes [9]. Both homologous and illegitimate recombination may occur at the *Glu-3* and *Glu-1* loci [9,39], resulting in the formation of novel allelic genes. In the current study, our results demonstrated that LMW-GS encoded by the *Glu-3* locus in *Brachypodium* also display the properties of a complex gene family as those in *Triticum* and related species. The numbers of copies of LMW-GS genes in *B. distachyon* are probably less than that in *T. aestivum* according to the Southern blotting analysis but similar mechanisms for generating allelic variations at the *Glu-3* locus might be present in *Brachypodium*. Frequent SNP and InDel variations, duplications and inversions of one and more repeats, by unequal crossing over, slippage or illegitimate recombination [9,40], could result in the allelic variations observed at *Glu-3* in *B. distachyon.*

### Phylogenetic evolutionary relationships of *B. distachyon* with *Triticum* and related species as revealed by *Glu-3* loci

*B. distachyon.* has been shown to be much more closely related to wheat, barley and rice than to sorghum, rye or maize [19,20]. Recent studies have shown that *B. distachyon* is closely related to the tribe Triticeae and *Ae. tauschii*, the donor of D genome of hexaploid wheat [18,41]. The analysis of bacterial artificial chromosome (BAC) end sequences (BES) of *Brachypodium* genome also revealed a closer relationship between *Brachypodium* and Triticeae than *Brachypodium* and rice or maize [42]. In the present work, phylogenetic tree based on the LMW-m type genes (Figure 5) indicated that *Brachypodium* is more closely related to *Aegilops* than to wheat, especially much closer to *Ae. markgrafii* (CC), *Ae. umbellulata* (UU), *Ae. uniaristata* (NN) and *Ae. tauschii* (DD). On the other hand, results revealed by the LMW-i type genes demonstrated that *B. distachyon* was closer to hexaploid common wheat and *Ae. markgrafii* than to *Ae. uniaristata* and *Ae. comosa*. It has been argued that the LMW-m type genes could be the progenitor of LMW-i and LMW-s type genes [32]. The HQ220202 and HQ220204 from *B. distachyon* and GQ870241 from the U genome of *Ae. umbellulata* were clustered into a clade while GQ870250 from the C genome of *Ae. markgrafii* had higher similarity with GQ870241. These 4 LMW-GS genes all had 9 cysteine residues located at a highly conserved position (Figure 4), indicating that *B. distachyon* is more closely related to *Ae. markgrafii* and *Ae. umbellulata*. Our results further supported the recent report that the C and U genomes appear to be closely related [32].

### Evolution of *Glu-1* and *Gli-1* loci in *B. distachyon*

Our work demonstrated that *B. distachyon* grains had similar compositions of albumins, globulins, and LMW-GS with these of wheat, but with fewer gliadins and HMW-GS. This suggests that, although the *Glu-3* loci are highly conserved among *Brachypodium*, *Aegilops*, *Triticum* and other related cereal species, the *Glu-1* and *Gli-1* loci in *B. distachyon* could undergo dramatic divergence during its evolutionary process. A recent report has shown that a single copy of HMW glutenin gene with a premature stop codon was found in *Brachypodium*, and its structure was considered to be different from the wheat HMW glutenin gene [43]. However, we identified a HMW glutenin subunit with a lower expression level in Bd21 by a proteomic approach [25] and its encoding gene has been recently cloned in our lab (data not shown). This suggests that the *Glu-1* is also conserved in *Brachypodium*.

In common wheat, LMW-GS are encoded by the genes at the orthologous *Glu-A3*, *Glu-B3* and *Glu-D3* loci on the short arms of group 1 chromosomes (1AS, 1BS and 1DS), which are closely linked with the *Gli-A1*, *Gli-B1* and *Gli-D1* loci encoding gliadins [44]. The physical relationships of *Glu-3* and *Gli-1* genes showed that gliadin or gliadin-like genes can distribute between two typical LMW-GS genes [45] and different types of LMW-GS can locate separately [9,38]. This can potentially result in formation of different chimeric genes between gliadin and the LMW-GS genes due to crossing over and illegitimate recombination between or in the *Gli-1* and *Glu-3* loci in wheat and related species [9,46]. Therefore, some modified LMW-GS may also be present in *Brachypodium* as reported in wheat [30].

According to our results, *B. distachyon* grains had very few gliadins when separated by the same extraction method as for wheat. Larré *et al*. also only found a few putative avenin-like proteins in *B. distachyon*[24]. We speculate that most of the gliadins in *B. distachyon* may have evolved into LMW-GS, most likely the LMW-C subunits. This may explain why *Brachypodium* contained abundant LMW-GS especially the LMW-C type subunits than wheat. Recent reports strongly support this scenario: some modified LMW-GS in wheat were identified by 2-DE and MALDI-TOF-MS, which might belong to modified α/β- and γ-gliadins [30]. It is also possible that most of the gliadin genes were pseudogenes and thus silent in mature grains due to premature stop codons as those in wheat, rice, maize and other cereals [47,48].

Both globulin and glutenin genes are specifically expressed in seed developing tissues. Particularly, glutenin genes have a higher expression level than globulin genes [43,49]. Since fewer HMW-GS and no gliadins are expressed in the grains, the LMW glutenin subunits with higher expression level could be important grain storage protein in *Brachypodium*.

## Conclusion

*Brachypodium* was more closely related to *Ae. markgrafii* and *Ae. umbellulata* than to *T. aestivum*; it possesses a highly conserved *Glu-3* locus. The presence of LMW-GS in *B. distachyon* t warrants its usage as a model plant system for wheat quality research*.*

## Methods

### Plant materials

Six *B. distachyon* accessions were used in this work, including 5 hexaploid genotypes (2n=6x=30): Bd4 (PI208216), Bd10 (PI226452), Bd11 (PI226629), Bd13 (PI233228) and Bd16 (PI239715), and 1 diploid accession Bd21 (2n=10). All accession seeds were kindly provided by Dr. John Vogel, USDA-ARS, Albany, CA and Dr. Chengtao Lin, Institute of Crop Sciences, Chinese Academy of Agricultural Sciences (CAAS). Hexaploid common wheat varieties (*Triticum aestivum* L.) Chinese Spring (CS), and Kontrast were used as control in the study.

### Grain protein extraction, SDS-PAGE, RP-HPLC, Western blotting and Southern blotting

*B. distachyon* seeds (50mg) were ground to powder and used to extract different grain proteins. The albumins, globulins and gliadins were extracted by distilled water, dilute salt solutions and 70% ethanol, respectively. The method of glutenin extraction and SDS-PAGE were adopted from Yan *et al*. [50]. The total albumins and globulins were extracted from 50 mg *B. distachyon* grains according to Gao *et al.*[26]. Same volume (15μl) for each sample was loaded per lane of the gels. SDS-PAGE was performed with a Bio-Rad PROTEAN II XL based on the previously described method [50]. Reverse-phase high performance liquid chromatography (RP-HPLC) was performed on the basis of the method of Gao *et al*. [51]. A polyclonal antibody (CIPGLERPWQQQPL) specific for wheat LMW glutenin subunits was developed for Western blotting analysis and the detailed procedures were according to Li et al. [52]. The procedures of Southern blotting were according to Yue *et al*. [53] with minor modifications. Total genomic DNA was isolated from young leaves of Bd21 using a modified cetyl-trimethyl- ammonium bromide protocol [54] and was quantified after RNase treatment. Considering that there were no *BamHI*, *EcoRI*, and *HindIIII* restriction sites in LMW-GS genes, the genomic DNA were digested with the three enzymes. The digested products were electrophoresed using 0.8% agarose gel and blotted onto a Hybond-N+ nylon membrane (Amersham, Buchinghamshire, UK) with alkaline transfer buffer (0.4 M NaOH). The PCR production of LMW-GS gene (FJ615311) from Chinese Spring were used as probes and the positive plasmid of LMW-GS gene was used as amplified template in above PCR system. Probe labeling, hybridization and detection of the LMW-GS genes were performed using the DIG High Prime DNA Labeling and Detection Starter Kit II (Roche, Mannheim, Germany), following the instructions of manufacturer. Four post-hybridization washes were performed as following: (1) Wash 2 x 5 min in ample 2 x SSC, 0.1% SDS at 25°C under constant agitation. (2) Wash 2 x 15 min in 0.5 x SSC, 0.1% SDS (prewarmed to wash temperature) at 68° Cunder constant agitation.

### DNA extraction and PCR amplification

Genomic DNA was extracted from 30mg leaves of *Brachypodium* seedlings 7 days after germination with cetytrimethylammonium bomide (CTAB) method as reported by Murray and Thompson [54]. Two pair of primers (Primer 1+2 and primer 7+8) as described by Pei *et al*. [6] and Jiang *et al*. [55] for amplifying wheat LMW-GS genes were used to amplify the complete open reading fragments (ORFs) of the LMW glutenin genes of *B. distachyon*. PCR reaction in a 30 μl volume was performed using a S1000^™^ thermal cycler (Bio-Rad, USA) with the following program: an initial step of 94°C for 4 min, 34 cycles of 94°C for 45 s, 58°C for 1 min and 72°C for 80 s, and a final step of 10 min at 72°C. The recombined DNA clones were sequenced by TaKaRa Biotech Inc., Japan. Each clone was sequenced three times to avoid possible error.

### Sequence comparison and SNPs/InDels identification

Bioedit 7.0 was used to conduct complete multiple alignment based on the complete nucleotide of cloned LMW-GS genes and other genes from GenBank. Single-nucleotide polymorphisms (SNPs) and insertions/deletions (InDels) among LMW glutentin genes from *B. distachyon* as well as *Aegilops* and *Triticum* species were identified based on the multiple alignments [10].

### MALDI-TOF-MS

The deduced proteins of cloned LMW-GS genes were further confirmed by matrix-assisted laser desorption/ionization time-off mass spectrometry (MALDI-TOF-MS). Based on the deduced amino acid sequences of LMW-GS genes cloned in this work, the theoretical mass spectra of trypsin digested LMW-GS were predicted by using the bioinformatic program PeptideMass (http://www.expasy.ch/tools/pep-tide-mass. html). 5 protein bands (4–1, 21–1, 21–2, 13–1 and 10–1) on SDS-PAGE gels (Figure 1e), corresponding to deduced molecular mass of cloned genes were selected for MS analysis. Protein bands were excised manually and their in-gel trypsin digestions were performed according to Gao *et al*. [26]. MALDI-TOF-MS identifications were conducted by searching against the NCBInr databases through the MASCOT (http://www.matrixscience.com) search engine and the putative *Brachypodium* protein database (http://www.brachypodium.org) [13]. Finally, a comparative analysis between predicted and MS detected mass spectra was performed.

### Phylogenetic analysis

MEGA4.1 was used to construct phylogenetic trees with complete nucleotide coding sequences of LMW-GS and the detailed steps were based on Wang *et al*. [32].

## Authors’ contributions

SW participated in most of the experiments and data analysis, including experimental designs, protein extraction and separation, evolutionary analysis, etc. KW did Southern-blotting, GC took part in experiment of Western-blotting and XH participated in cloning the genes. ZY and XL did RP-HPLC. DL took part in some work of analyzing the result of LMW-GS mass spectrum. YY, XY and WM took part in designing and supervising the study. SW, YY and WM participated in drafting the manuscript. SLK Hsam, R Appels gave valuable advice on manuscript preparation. All authors have read and approved the final manuscript.

## Supplementary Material

Additional file 1 **PCR amplification products on agarose gel of 6*B. distachyon*accessions.** The samples of lane 1–6 from left to right are *Brachypodium* accession Bd4 (PI208216), Bd10 (PI226452), Bd11 (PI226629), Bd13 (PI233228), Bd16 (PI239715) and Bd21. M represents 1Kb DNA marker, a. PCR amplification results by primer 1 and 2, b. PCR amplification results by primer 7 and 8. Click here for file

Additional file 2 **MALDI mass spectrum of the tryptic peptides of the protein band 4–1 from Bd4 which was markedin Figure**1. Click here for file

Additional file 3 **MALDI mass spectrum of the tryptic peptides of the protein band 22–1 from Bd21 which was marked in Figure**1. Click here for file

Additional file 4 **MALDI mass spectrum of the tryptic peptides of the protein band13–1 from Bd13 which was marked in Figure**1. Click here for file

Additional file 5 **MALDI mass spectrum of the tryptic peptides of the protein band 10–1 from Bd10 which was marked in Figure**1. Click here for file

Additional file 6 **MALDI mass spectrum of the tryptic peptides of the protein band 21–2 from Bd21 which was marked in Figure**1. Click here for file

Additional file 7 **Protein identification of 21–2 in*B. distachyon*by MALDI-TOF-MS.** Click here for file
