# Supplementary figures and images for: Molecular characterization of LMW-GS genes in Brachypodium distachyon L. reveals highly conserved Glu-3 loci in Triticum and related species
Source: BMC Plant Biol. 2012 Nov 21;12:221. doi: 10.1186/1471-2229-12-221 (PMC3547698; doi:10.1186/1471-2229-12-221)

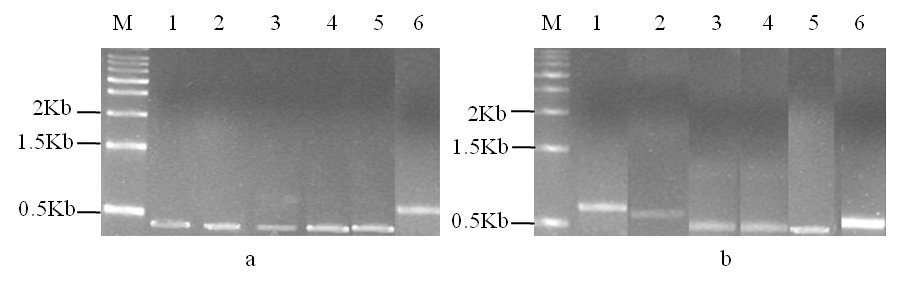

Supplement: Additional file 1 — PCR amplification products on agarose gel of 6B. distachyonaccessions. The samples of lane 1–6 from left to right are Brachypodium accession Bd4 (PI208216), Bd10 (PI226452), Bd11 (PI226629), Bd13 (PI233228), Bd16 (PI239715) and Bd21. M represents 1Kb DNA marker, a. PCR amplification results by primer 1 and 2, b. PCR amplification results by primer 7 and 8. [file 1471-2229-12-221-S1.png]

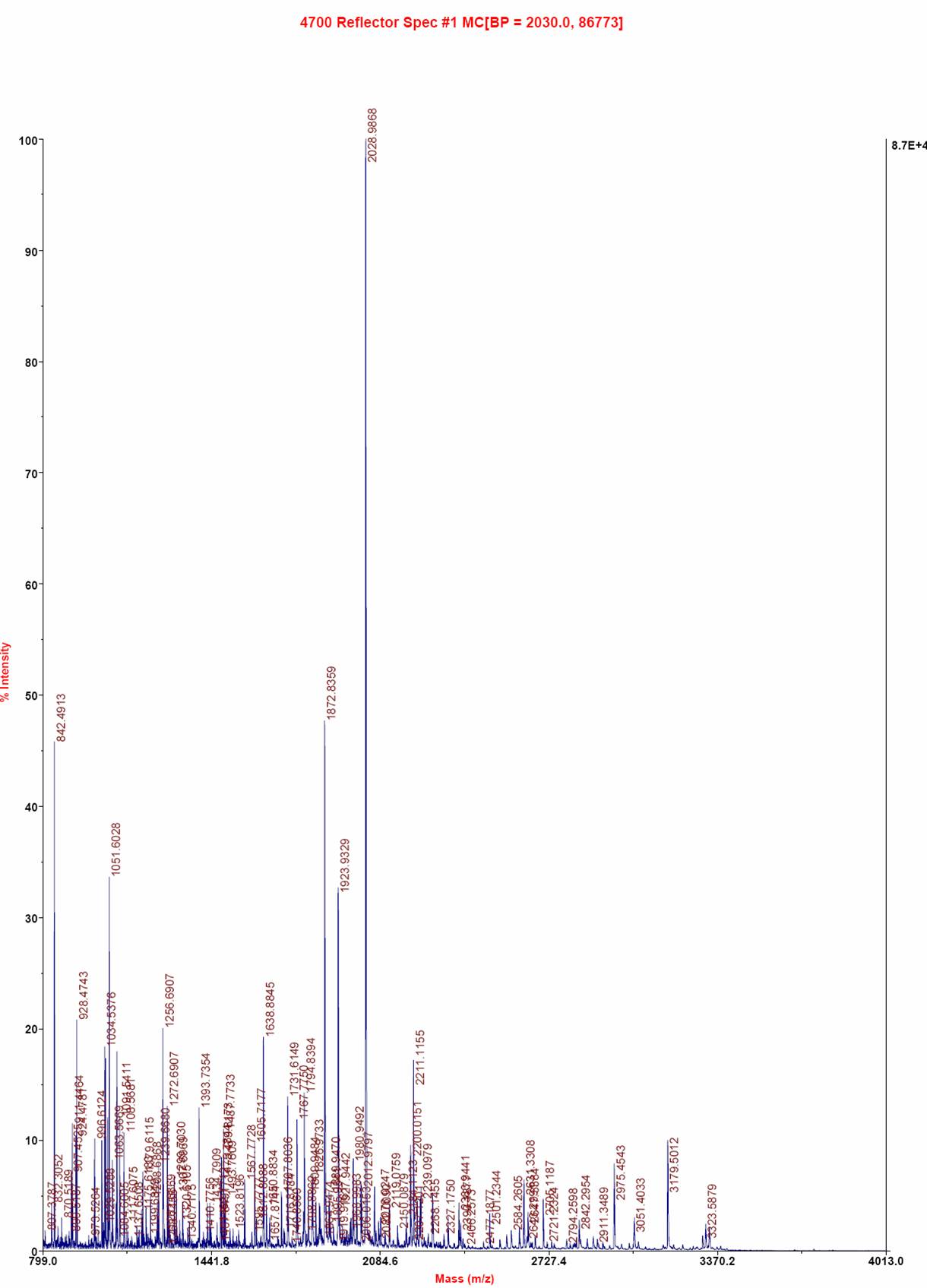

Supplement: Additional file 2 — MALDI mass spectrum of the tryptic peptides of the protein band 4–1 from Bd4 which was markedin Figure1. [file 1471-2229-12-221-S2.jpeg]

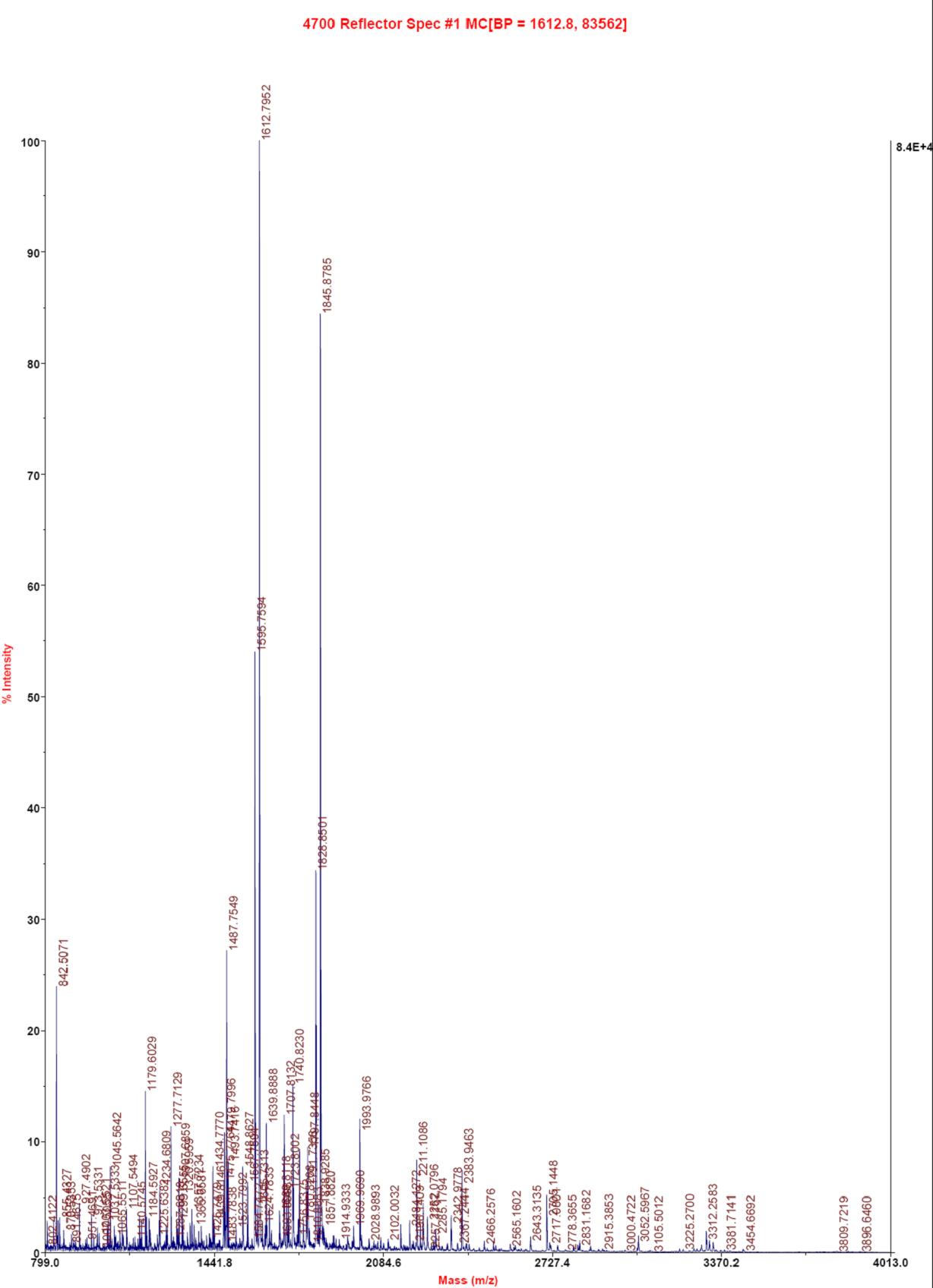

Supplement: Additional file 3 — MALDI mass spectrum of the tryptic peptides of the protein band 22–1 from Bd21 which was marked in Figure1. [file 1471-2229-12-221-S3.jpeg]

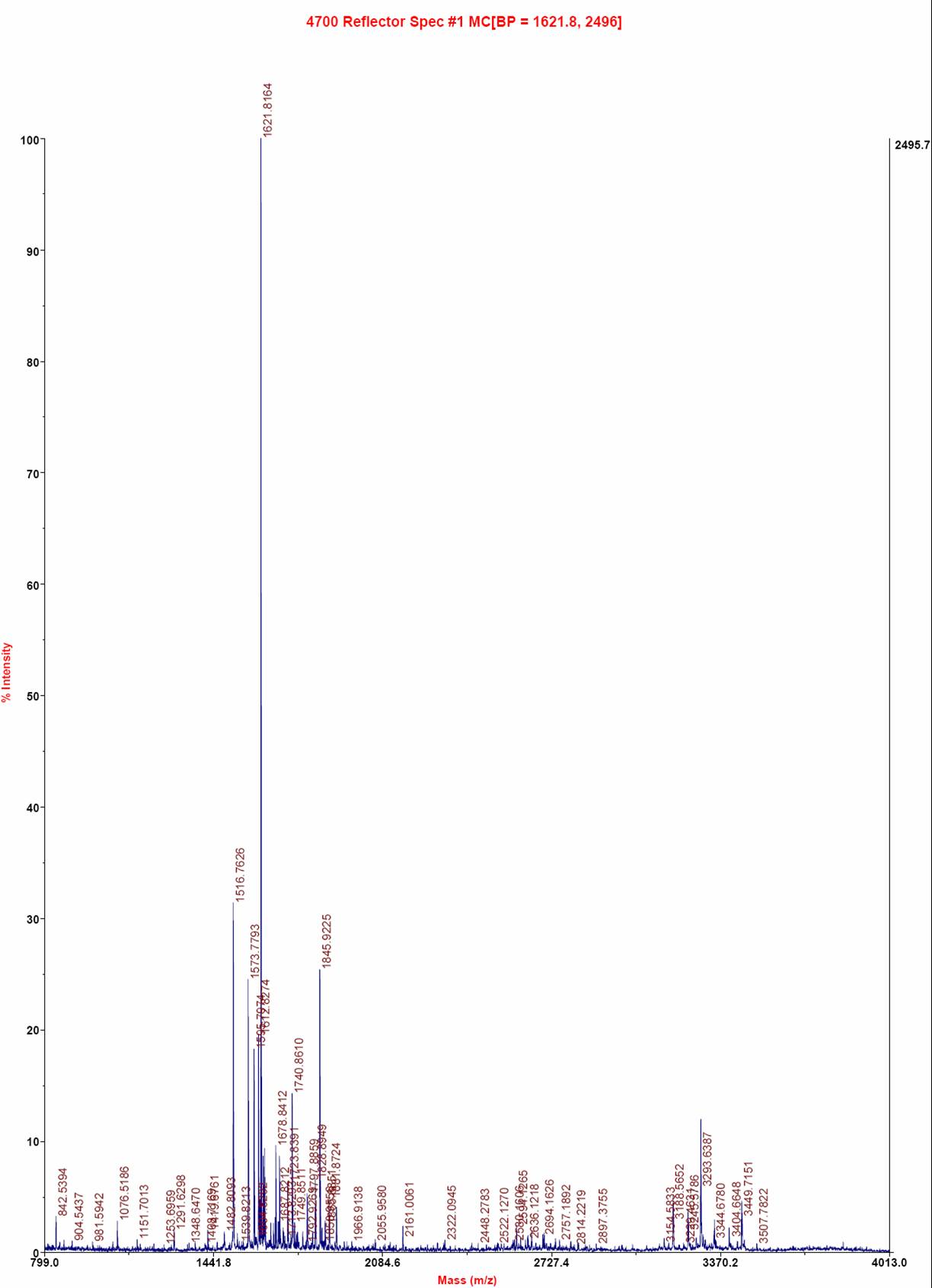

Supplement: Additional file 4 — MALDI mass spectrum of the tryptic peptides of the protein band13–1 from Bd13 which was marked in Figure1. [file 1471-2229-12-221-S4.jpeg]

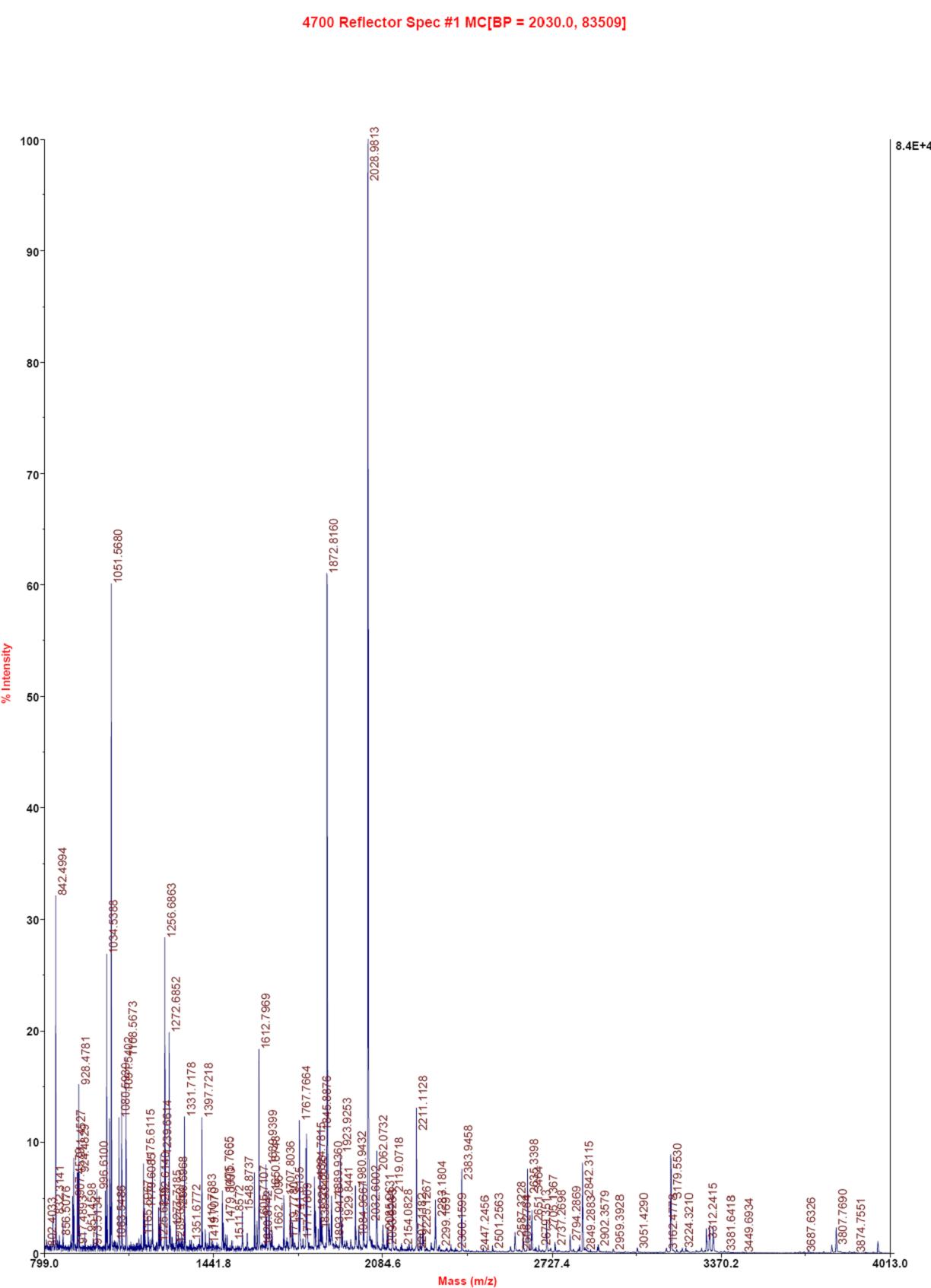

Supplement: Additional file 5 — MALDI mass spectrum of the tryptic peptides of the protein band 10–1 from Bd10 which was marked in Figure1. [file 1471-2229-12-221-S5.jpeg]

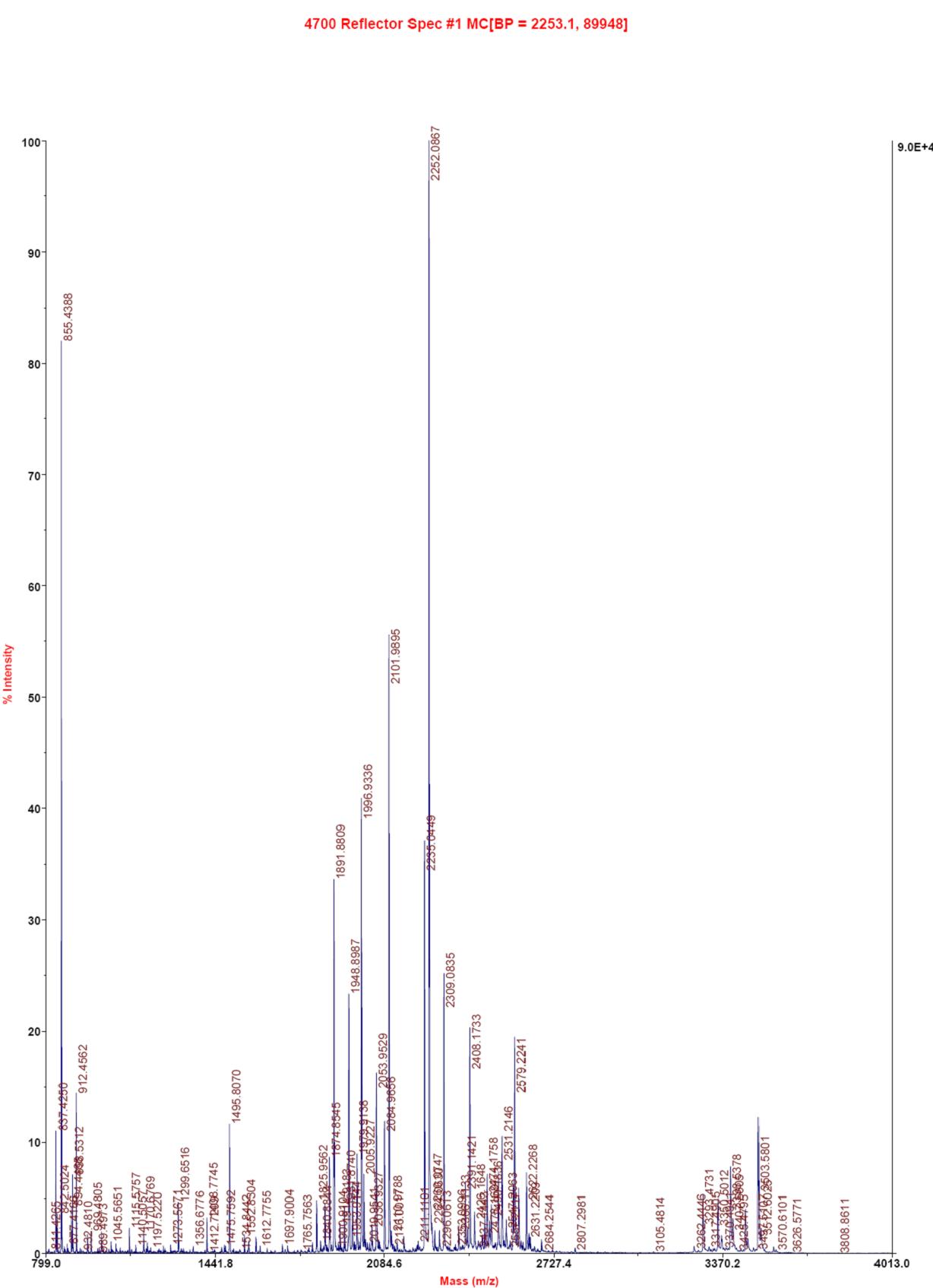

Supplement: Additional file 6 — MALDI mass spectrum of the tryptic peptides of the protein band 21–2 from Bd21 which was marked in Figure1. [file 1471-2229-12-221-S6.jpeg]
