# Supplementary material for: Molecular characterization of LMW-GS genes in Brachypodium distachyon L. reveals highly conserved Glu-3 loci in Triticum and related species
Source: BMC Plant Biol. 2012 Nov 21;12:221. doi: 10.1186/1471-2229-12-221 (PMC3547698; doi:10.1186/1471-2229-12-221)
Supplement: Additional file 7 — Protein identification of 21–2 inB. distachyonby MALDI-TOF-MS. [file 1471-2229-12-221-S7.doc]

| Band Name | Protein identified | Plant species | gi Number | Protein Score | Tryptic Fragments identified by MS/MS | Calculate Mass | Measured mass | positions |
| --- | --- | --- | --- | --- | --- | --- | --- | --- |
| 21-2 | prolamine | *B. distachyon* | 193848597 | 126 | QQQQQQGQAQSTPR | 1612.7787 | 1612.7941 | 258-271 |
|  |  |  |  |  | MKTFLILALLALVMATTASAR | 2235.2966 | 2235.1648 | 1-21 |
